# Panel of miR-150 and linc00673, regulators of CCR6/CCL20 may serve as non-invasive diagnostic marker of non-small cell lung cancer

**DOI:** 10.1038/s41598-023-36485-7

**Published:** 2023-06-14

**Authors:** Kamila Baran, Jacek Kordiak, Sławomir Jabłoński, Ewa Brzeziańska-Lasota

**Affiliations:** 1 Department of Biomedicine and Genetics, Chair of Biology and Medical Microbiology, Medical University of Lodz, Lodz, Poland; 2 Department of Thoracic, General and Oncological Surgery, Medical University of Lodz, Lodz, Poland

**Keywords:** Cancer, Immunology, Molecular biology, Biomarkers, Oncology

## Abstract

The C–C motif ligand 20 (CCL20) is a chemokine that specifically binds to the chemokine receptor 6 (CCR6) and the CCL20/CCR6 axis has been implicated in the non-small lung cancer (NSCLC) development and progression. Its expression is regulated by mutual interactions of non-coding RNAs (ncRNAs). This goals of presented study was to evaluate the expression level of *CCR6/CCL20* mRNA in NSCLC tissue comparative to selected ncRNAs: miR-150, linc00673. The expression level of the studied ncRNAs was also assessed in serum extracellular vesicles (EVs). Thirty patients (n = 30) were enrolled as the study cohort. Total RNA was isolated from tumor tissue, adjacent macroscopically unchanged tissue and serum EVs. The expression level of studied genes and ncRNAs were estimated based on the qPCR method. Higher expression level of *CCL20* mRNA but lower expression level of *CCR6* mRNA were observed in tumor in comparison to control tissue. Relative to the smoking status, higher *CCL20* (p < 0.05) and *CCR6* mRNA (p > 0.05) expression levels were observed in current smokers than in never smokers. In serum EVs the expression level of miR-150 has a negative correlation with AJCC tumor staging, whereas the expression level of linc00673 positively correlated (p > 0.05). The lower expression level of miR-150 and higher expression level of linc00673 in serum EVs were observed in NSCLC patients with lymph nodes metastases (p > 0.05). Regarding the histopathological type, significantly lower expression level of miR-150 and higher expression level of linc00673 were observed in the serum EVs of patients with AC compared to patient with SCC. Our findings revealed that smoking significantly changed the expression level of *CCL20* mRNA in NSCLC tissue. Changes in expression levels of miR-150 and linc00673 in the serum EVs of NSCLC patients in relation to presence of lymph node metastases and the stage of cancer development may serve as a non-invasive molecular biomarkers of tumor progression. Furthermore, expression levels of miR-150 and linc00673 may serve as non-intrusive diagnostic biomarkers differentiating adenocarcinoma from squamous cell carcinoma.

## Introduction

Lung cancer is the most commonly diagnosed malignant neoplasm among men, and the third most frequent cancer affecting women worldwide^1^. According to the latest data released by WHO, 2 206 771 new cases of lung cancer were diagnosed in 2020, which poses 11.4% of all cancer cases^1^. Lung cancer is divided into two histological type: small lung cancer (SCLC) and non-small cancer (NSCLC). The most frequent diagnosed type of lung cancer is NSCLC, which, unfortunately, is characterized by a poor 5-year survival rate (26%)^2^. The high mortality of this cancer is mainly related to too late diagnosis of patients who are already in an advanced stage of the disease. Understanding molecular mechanisms in charge of cancer development and progression may be helpful in the development of diagnostic markers and new therapeutic targets.

Cigarette smoking is a vital risk factor conducive to the development of lung cancer and it is estimated that 80–90% of cases are related to this addiction^3^. Cigarette smoke causes inflammation in the lungs and is a factor that initiating the secretion of inflammatory cytokines, such as tumor necrosis factor (TNF), transforming growth factor-β (TGF-β), interleukin-1 (IL-1), IL-6, IL-8, and chemokines, including C-X-C motif ligand 9 (CXCL9), C-X-C motif ligand 10 (CXCL10) and also C–C motif ligand 20 (CCL20)^4,5^. An in vitro study conducted by Wang et al. revealed that nicotine-derived nitrosaminoketone (NNK; or tobacco-specific nitrosamine 4-(methylnitrosamino)-1-(3-pyridyl)-1-butanone), being a key carcinogen of tobacco smoke, significantly increases expression of CCL20 at protein and mRNA level in normal human lung epithelial cells^5^. CCL20, known also as macrophage inflammatory protein (MIP)-3α, is a specific ligand that activates C–C chemokine receptor 6 (CCR6)^6^. After binding of this ligand with the receptor, intracellular signaling pathways are activated, including ERK1/2-MAPK, Wnt and PI3K pathways, which are responsible for an increase in the proliferation of NSCLC cancer cells, enhance their migration rate and induce the epithelial-mesenchymal transition (EMT)^5–7^. However, it was also revealed that CCL20 may play a protective role and inhibit NSCLC tumor growth^8,9^. The studies showed that this chemokine stimulates the migration of immune cells, that possess CCR6, such as B cells, T cells (particularly Th17 cells and Treg cells), natural killer T (NKT) cells, neutrophils and immature dendritic cells (DC) to the tumor site, which suppress its development^8,9^. Hence, the function of the CCR6/CCL20 axis in the development of lung tumor is highly complex.

Cancer biology research should focus not only on the involvement of protein-coding genes in tumorigenesis, but also on the role of non-coding protein sequences, making up 97% of the human genome^10^. The most exhaustive researches have been conducted to investigate the role of micro-RNAs (miRNAs) in cancer development. miRNAs are involved in post-transcriptional gene regulation through translation repression or mRNA degradation, depending on miRNA-mRNA complementarity, while lncRNAs regulates gene expression through interaction domains for DNA, mRNA, miRNA and proteins, according to their sequence and secondary structure^11^. The tissue-specific expression pattern of miRNAs and lncRNAs is highly dysregulated during cancer development, therefore they are hopeful diagnostic, and therapeutic targets^12–14^. However, the exact function and mechanism of its action of most of them is still unexplained.

MicroRNA-150 has been considered a significant molecule involved in tumor initiation and progression of various cancers. However, its importance in NSCLC still remains controversial^15–18^. A study of Zhang et al. revealed that miR-150 is engaged in regulation of cell cycle and confirmed the anti-apoptotic function of this miRNA due to inhibition of the p53 protein expression level^16^. In turn, Gu et al. demonstrated that miR-150 affects the cell cycle of NSCLC cells by inhibiting the expression level of *BAK1*, coding an important pro-apoptotic regulator human BRI1-associated receptor kinase 1 (BAK1)^17^. Moreover, Li et al. confirmed in their study that this molecule is vital pro-metastatic miRNA because it plays an important role in regulation of migration and EMT^15^. However, contrary to these findings, a study of Dai et al. showed that miR-150 is characterized with a suppressive function in NSCLC as it inhibits Wnt signaling pathway^18^. They also revealed a low expression level of miR-150 in NSCLC patients correlates with a shorter progression-free time, overall survival time and a higher relapse rate and may be served as a potential biomarker for predicting NSCLC progression. Recently published data show that lncRNAs can interact with miRNAs^19^. It was recently revealed that long non-coding RNAs (lncRNAs) can pose endogenous competitors of miRNAs, and serve as specific “miRNA sponges” by possessing miRNA binding sequences, which results in blocking its function^20,21^.

A study by Lu et al. demonstrated that the expression level of miR-150 can be reduced during tumorigenesis by its binding to long intergenic non-coding RNA 673 (linc00673)^22^. Linc00673 contains a conserved region that is greatly similar to steroid receptor RNA activator 1 (SRA1) and due to this fact it is also named “SRA-like non-coding RNA” (SLNCR)^23^. In vitro studies identified linc00673 as an oncogene, which enhances cell proliferation, migration, invasion and complex epithelial-mesenchymal transition (EMT) in NSCLC cell lines by activation of TGF-β signaling pathway^22,24,25^. Among tumor promoting mechanisms of NSCLC, in which linc00073 is involved, the modification of histone H3K4, which causes epigenetic silencing of the suppressor gene NCALD, has been confirmed^25^. Furthermore, linc00673 can bind to the 3' untranslated region (UTR) of *TP53* mRNA reducing its expression level which lead to inhibition of p53-mediated cell cycle arrest^24^.

The aim of present study was to assess changes in both the expression levels of *CCR6* and *CCL20* in tumor tissue of NSCLC patients and the expression level of miR-150, that is known to regulate the expression level of *CCR6* according to data-base mirTarBased. The expression level of linc00673 in tumor tissue, which may influence the expression level of miR-150, was also determined. It should be pointed out that dysregulation in the expression level of ncRNA in liquid biopsy may be non-invasive biomarkers of NSCLC development and progression. Therefore, in our study we also analyzed differences in the expression level of studied ncRNAs not only in tumor tissue, but also in the serum EVs of NSCLC patients in relation to selected clinical features.

## Materials and methods

### Patients’ clinical characteristics

The study cohort involved of 30 patients (n = 30) with a confirmed diagnosis of NSCLC, 13 women and 17 men, aged 55 to 79 (mean age 67 ± 6.38 years), who underwent lung resection (pulmonectomy or lobectomy) at the Department of Thoracic Surgery, General and Oncological Surgery, Military Medical Academy Memorial Teaching Hospital of the Medical University of Lodz—Central Veterans’ Hospital, Lodz, Poland during the years 2018–2019. Serum samples were obtain before surgery from all classified NSCLC patients after excluding patients with no informed consent, too small tumor volume or preoperative neoadjuvant therapy. The smoking history and status were available for all patients. Patients were divided into groups depending on the members of pack-years (PYs), 1 pack-year means 20 cigarettes smoked per day for 1 year^26^. The clinical features, smoking status and history of the patients with diagnosed NSCLC are shown in Table 1.

**Table 1:** Clinical characteristics and smoking history of NSCLC patients.

Patient characteristics	Number of patients (n)	Total percentage of patients
Gender		
Female	13	43.33%
Male	17	56.67%
Age		
≤ 65	12	40%
> 65	18	60%
Smoking status		
Never smokers	8	26.67%
Former smokers	11	36.37%
Current smokers	11	36.37%
Smoking history		
≤ 35 PYs	12	40%
> 35 PYs	10	33.33%

*PYs* pack years.

### Materials collection

Tissue fragments from the primary tumor of NSCLC and adjacent macroscopically-unchanged tissue fragments, using as control, were obtained from thirty NSCLC patients (n = 30). Serum specimens ware collected from all NSCLC patients before undergoing the surgery and from 15 volunteers (n = 15) without cancer, which served as a control. All collected biological material was secured and prepared according to the protocol described in the our previous article^27^. NSCLC patients were not treated with chemo- or radiotherapy. Tissue samples were post-operatively histopathologically evaluated and classified according to the TNM and AJCC grading systems^28^. Clinical and histopathological evaluation of biological materials is demonstrated in Table 2.

**Table 2:** Clinical and histopathological characteristics of NSCLC tissue samples.

Tumor features	Number of cases (n)	Total percentage of cases
Histopathological subtype of NSCLC		
SCC	13	43.33%
AC	15	50%
LCLC	2	6.67%
Stage of cancer development according to AJCC staging system		
Stage I	11	36.37%
Stage II	12	40%
Stage III	7	23.33%
Presence of lymph node metastasis according to the pTNM staging system		
N0	18	60%
N1 + N2	12	40%
Size of tumor according to the pTNM staging system		
T1a + T1b	9	30%
T2a + T2b	12	40%
T3 + T4	9	30%

*SCC* squamous cell carcinoma, *AC* adenocarcinoma, LCLC large cell lung cancer, *AJCC* American Joint Committee on Cancer Staging according to the IASCLC Staging Project 7th ed. (2010) Cancer, *pTNM* post-operative Tumor Node Metastasis staging system according to the WHO Histological Typing of Lung Tumor.

### RNA isolation from tissue and serum EVs, qualitative and quantitative RNA evaluation

Total RNA from tissue homogenates was isolated with the use of the mirVana™ miRNA Isolation Kit (Life Technologies, Carlsbad, CA, USA) according to the manufacturer’s protocol. EVs from the serum were extracted using the Total Exosome Isolation Reagent. Total Exosome RNA was extracted from exosomes & Protein Isolation Kit (Applied Biosystems, USA), according to the manufacturer’s instructions.

Qualitative and quantitative assessment of the RNA was conducted by the spectrophotometric method (260/280 nm) with an Eppendorf BioPhotometerTM Plus apparatus (Eppendorf, Hamburg, Germany).

### Evaluation of relative expression level (RQ) of genes and ncRNAs

Reverse transcription (RT) for genes and lncRNA was performed with the use of the High-Capacity cDNA Reverse Transcription Kit (Applied Biosystems, USA), whereas the TaqMan^®^ MicroRNA Reverse Transcription Kit (Applied Biosystems, Carlsbad, CA) was used for miR-150 in accordance with the manufacturer’s protocol. RT reaction was performed in a Personal Thermocycler (Eppendorf, Germany).

The relative expression level analysis for studied genes and ncRNA was performed in 7900HT Fast Real-Time PCR System (Applied Biosystems, Carlsbad, CA, USA) using TaqMan assay: *CCR6* (Hs01890706_s1) *CCL20* (Hs00355476_m1), linc00673 (Hs05048495_s1), miR-150 (UCUCCCAACCCUUGUACCAGUG), as well as *ACTB* (Hs01060665_g1) and RNU6B (CGCAAGGATGACACGCAAATTCGTGAAGCGTTCCATATTTTT), which were used as an endogenous control for genes/lncRNA and miRNA, respectively.

The relative expression levels of the study genes and ncRNA in lung tissue were assessed by the delta–delta CT method (TaqMan Relative Quantification Assay software, Applied Biosystems). RNA from normal lung tissue (Human Lung Total RNA, Ambion^®^, USA) was used as calibrator and its expression level was regarded as RQ = 1.

The RQ values of the studied miRNA and lncRNA in serum EVs were evaluated with global normalization. The median CT of those assays is used as the normalizer, on a per sample basis.

All methods were performed in accordance with the relevant guidelines and regulations.

### Statistical analysis

The RQ values for the study genes and ncRNA were showed as median values. Due to the fact that distributions of most of the variables were significantly different from the normal distribution, which were assessed with Shapiro–Wilk test, we conducted non-parametric tests: the Mann–Whitney U test (for two-group comparisons), or the Kruskal–Wallis test (for multiple group comparisons). The Spearman rank correlation coefficient was used to measure the direction and strength of the association for individual variables. The area under the receiver operating characteristic (ROC) curve was assessed to test the sensitivity and specificity of the studied ncRNAs. The area under the curve (AUC) was resolved with a 95% confidence interval (CI). All analyses were performed using the Statistica for Windows 10.0 software. The level of statistical significance was p < 0.05.

### Institutional review board statement

The Bioethics Committee of the Medical University of Lodz, Poland approved the study (resolution No. KE/149/18).

### Informed consent statement

All patients gave their informed consent to participate in the study.

## Results

### Relative expression level of the CCR6 and CCL20 mRNA in NSCLC tissue vs. control lung tissue

mRNA expression level of *CCR6* was downregulated (RQ < 1) in 3% of NSCLC tissue samples, while in all control tissue fragments (100%) its expression level was upregulated (RQ > 1). A lower expression level of *CCR6* mRNA was noticed in tumor tissue compared to the control tissue (median RQ: 7.813 and 12.408, respectively), but this difference was statistically insignificant (p > 0.05, Mann–Whitney U-test).

In relation to the histopathological type, a slightly higher expression level of *CCR6* mRNA was observed in tumor tissue of AC patients compared to control tissue (median RQ: 8.778 and 8.756, respectively) but the differences were statistically insignificant (p > 0.05, Mann–Whitney U-test). A statistically significant difference was observed in the expression level of *CCR6* mRNA between tumor tissue of SCC patients and control tissue (p = 0.044, Mann–Whitney U-test), with a lower *CCR6* mRNA expression level in tumor tissue (median RQ: 7.781 and 17.498, respectively).

*CCL20* mRNA expression level was upregulated (RQ > 1) in 47% of NSCLC tissue samples and in 23% of control tissue fragments. A higher expression level of *CCL20* mRNA was observed in tumor tissue than control tissue (median RQ: 0.726 and 0.369, respectively). The differences in the expression level of *CCL20* between study groups were statistically insignificant (p > 0.05, Mann–Whitney U-test).

The expression level of *CCL20* was higher in tumor tissue of AC patients than control tissue (median RQ: 2.399 and 0.411, respectively), as well as in tumor tissue of SCC patients compared to control (median RQ: 0.287 and 0.174, respectively); however, the differences were statistically insignificant (p > 0.05, Mann–Whitney U-test).

### Relative expression levels of CCR6 and CCL20 mRNA in NSCLC tissue according to biological characteristics and smoking history of study patients

The *CCR6* mRNA expression level in tumor tissue was lower in female patients compared to the male group (median RQ: 7.622 and 7.845, respectively), as well as in patients aged > 65 than in those aged ≤ 65 (median RQ: 12.954 and 10.634, respectively), but the differences were statistically insignificant (p > 0.05, Mann–Whitney U-test) (Table 3).

**Table 3:** Median expression level (RQ value) of studied genes and ncRNAs in NSCLC tissue according to clinical and pathological characteristics.

Clinical and pathological features	*CCR6* (median RQ)	p value	*CCL20* (median RQ)	p value	miR-150 (median RQ)	p value	linc00673 (median RQ)	p value
Gender								
Female	7.622	> 0.05	0.741	> 0.05	34.195	> 0.05	51.474	> 0.05
Male	7.845		0.710		18.684		40.107	
Age								
≤ 65	8.252	> 0.05	1.767	> 0.05	14.055	> 0.05	55.734	> 0.05
> 65	6.956		0.600		45.561		44.003	
Smoking status								
Never smokers	5.435	> 0.05	0.339	**0.041**	25.596	> 0.05	54.742	> 0.05
Former smokers	6.130		2.928		18.684		34.720	
Current smokers	13.750		0.565		69.173		59.524	
Smoking history								
≤ 35 PYs	8.220	> 0.05	1.574	> 0.05	23.410	> 0.05	52.220	> 0.05
> 35 PYs	9.0607		0.914		48.049		35.713	
Histopathological subtype								
SCC	7.781	> 0.05	0.287	**0.008**	22.322	> 0.05	60.564	> 0.05
AC	8.778		2.399		28.869		43.877	
AJCC staging system								
Stage I	5.851	> 0.05	0.741	> 0.05	34.596	> 0.05	51.474	> 0.05
Stage II	8.718		0.886		22.804		35.713	
Stage III	7.845		0.357		10.965		66.817	
pTNM (N)								
N0	7.701	> 0.05	1.127	> 0.05	45.361	> 0.05	58.767	> 0.05
N1 + N2	11.179		0.352		14.055		33.020	
pTNM (T)								
T1	15.970	> 0.05	0.741	> 0.05	34.195	> 0.05	59.524	> 0.05
T2	9.573		0.456		29.358		35.626	
T3 + T4	5.048		1.136		18.684		67.590	

Significant value is in bold.

Statistically significant p < 0.05.

The *CCL20* mRNA expression level was lower in males compared to females (median RQ: 0.710 and 0.741, respectively) but significant differences were not observed (p > 0.05, Mann–Whitney U-test) (Table 3). Statistically insignificant differences in *CCL20* mRNA expression level were noted in relation to age. A higher *CCL20* mRNA expression level was found in those aged ≤ 65 than > 65 (median RQ: 1.767 and 0.600, respectively) (Table 3).

Relative to the smoking status, the *CCR6* mRNA expression level was higher in current smokers compared to former smokers, as well as never smokers (median RQ: 13.750, 6.130 and 5.435, respectively), but there were statistically insignificant differences between these groups (p > 0.05, Kruskal-Willis test) (Table 3). In the group of patients who smoked > 35 PYs, the *CCR6* mRNA expression level was higher than in those who smoked ≤ 35 PYs (median RQ: 9.061 and 8.220, respectively). However, insignificant differences were noted (p > 0.05, Mann–Whitney U-test).

**Figure 1:**
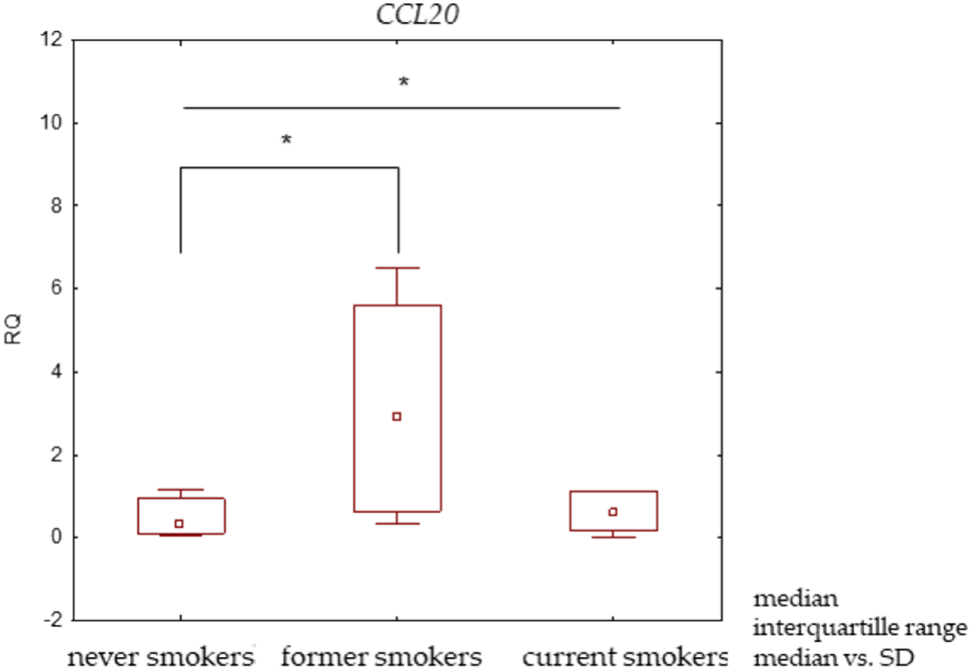
Box plot presenting differences in median RQ values for *CCL20* in NSCLC tissue according to the smoking status of patients, * p < 0.05.

A higher *CCL20* mRNA expression level was observed in current smokers than in never smokers. Yet, the level was lower than in former smokers (Table 3). Statistically significant differences were observed between these study groups (Fig. 1). Relative to smoking history, higher expression levels of *CCL20* mRNA were observed in patients who smoked ≤ 35 PYs than in those who smoked > 35 PYs; however, no significant differences were noted (Table 3).

### Relative expression levels of CCR6 and CCL20 mRNA in NSCLC tissue in relation to histopathological evaluation and the TNM /AJCC staging system

Relative to the histopathological type, higher *CCR6* and *CCL20* mRNA expression levels were observed in the AC group compared to the SCC group (Table 3). Statistically significant differences were observed between the expression level of *CCL20* mRNA and these two cancer types (p < 0.05, Mann–Whitney U-test), but no differences were noticed among the *CCR6* mRNA expression level.

Regarding the malignant stage (AJCC staging system), the highest expression level of study genes was observed in stage II. The lowest *CCR6* mRNA expression level was in stage I; however, the lowest expression level for *CCL20* was noted in stage III (Table 3). Statistically insignificant correlations were observed for the cancer stage (p > 0.05, Kruskal-Willis test).

With regard to involvement of lymph nodes (N feature of the pTNM classification), lower *CCR6* and *CCL20* mRNA expression levels were observed among patients with diagnosed lymph nodes metastases (N1 + N2), compared to those without them (N0). However, these differences were statistically insignificant (p > 0.05, Mann–Whitney U-test) (Table 3).

Statistically insignificant correlations were observed between *CCR6* and *CCL20* mRNA expression levels and tumor size (p > 0.05, Kruskal–Wallis test). The expression level of *CCR6* mRNA was the highest in patient with T1 and the lowest in patients with T3 + T4. In turn, the lowest *CCL20* mRNA expression was observed in those with T2 and the highest in patients with T3 + T4 (Table 3).

### Relative expression levels of miR-150 and linc00673 in NSCLC tissue vs. control lung tissue

An upregulated expression level of miR-150 (RQ > 1) was observed in 93% of tumor tissue and in all (100%) control tissue. In tumor tissue, the expression level of miR-150 was significantly lower compared to control tissue (median RQ: 27.897 and 336.319) (p = 0.00005, Mann–Whitney U-test). All tumor tissue and control tissue an showed upregulated expression level of linc00673. Statistically insignificant differences were noticed in the expression level of linc00673 in tumor tissue than control; however, its expression level was higher in tumor tissue (median RQ: 47.675 and 23.477, respectively).

**Figure 2:**
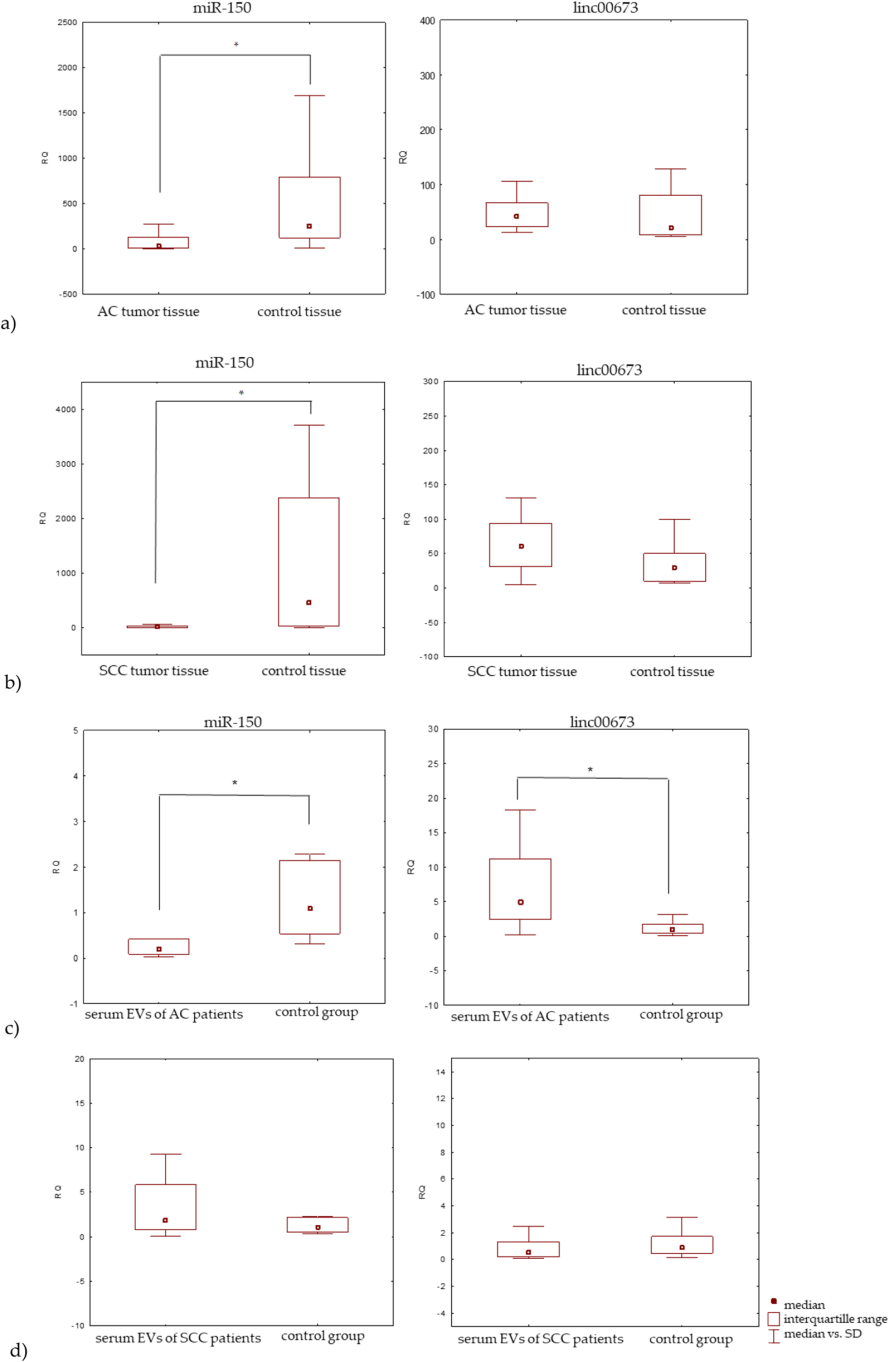
Box plot presenting differences in median RQ values for miR-150 and linc00673 in: (**a**) tumor tissue vs. control tissue of AC patients, (**b**) tumor tissue vs. control tissue of SCC patients, (**c**) serum EVs of AC patients vs. control group, (**d**) serum EVs of SCC patients vs. control group; * p < 0.05.

In relation to the histopathological type, the expression level of miR-150 was significantly lower in AC tumor tissue compared to control tissue (median RQ: 28.869 and 248.690, respectively), and also in SCC tumor tissue its expression level was significantly lower than in control (median RQ: 22.322 and 448.282, respectively) (Fig. 2). Conversely, the expression level of linc00673 was higher in AC tumor tissue and also in SCC tumor tissue compared to control tissue (median RQ: 43.877, 20.160 and 60.564 and 28.170, respectively). Statistically insignificant differences were found between expression of lncRNA and study groups (p > 0.05, Mann–Whitney U-test) (Fig. 2).

### Relative expression levels of miR-150 and linc00673 in NSCLC tissue according to biological characteristics and smoking history of study patients

The lower expression level of miR-150 and higher expression level of linc00673 were observed in the group of women compared to the group of men (Table 3); however, no significant differences were found between the study groups (p > 0.05, Mann–Whitney U-test).

In patients aged > 65 observed a higher miR-150 expression level and lower linc00673 expression level compared to those aged 65 years and younger (Table 3); however, the differences were statistically insignificant (p > 0.05, Mann–Whitney U test).

Relative to the smoking status, the miR-150 and linc00673 expression level was lower in never smokers compared to current smokers (median RQ: 25.596, 54.742 and 69.173, 59.524, respectively), but higher than former smokers (median RQ: 18.684 and 34.720, respectively) (Table 3). All observed differences in expression level were not statistically significant (p > 0.05, Kruskal-Willis test). In the group of patients who smoked > 35 PYs, the expression level of miR-150 was higher than in those who smoked ≤ 35 PYs (median RQ: 48.049 and 23.410, respectively). In contrast, a lower expression level of linc00673 was observed in the group of patients who smoked > 35 PYs compared to those who smoked ≤ 35 PYs (median RQ: 35.713 and 52.220, respectively). Statistically insignificant differences were noted with regard to smoking history (p > 0.05, Mann–Whitney U-test) (Table 3).

### Relative expression levels of miR-150 and linc00673 in NSCLC tissue in relation to histopathological evaluation and the TNM /AJCC staging system

Statistically insignificant differences in miR-150 and linc00673 expression levels were observed regarding to the histopathological evaluation (p > 0.05, Mann–Whitney U-test). In SCC, miRNA-150 expression level was decreased but linc00673 expression level was elevated (Table 3).

With regard to the AJCC staging system, the highest miR-150 expression level was noticed in a patient with stage I and the lowest in stage III (Table 3). In turn, patients with stage I, demonstrated the highest linc00673 expression level (median RQ was 66.817). However, its expression level was higher in patients with stage I than in stage II patients (median RQ: 51.474 and 35.713, respectively). Statistical differences but not significant in miR-150 and linc00673 expression levels were noticed with regard to AJCC stage (p > 0.05, Kruskal-Willis test) (Table 3).

Statistically insignificant differences in miR-150 and linc00673 expression levels were observed in tissues of patients with NSCLC with regards to lymph node involvement—N feature (N0, N1 + N2) according to the pTNM classification (p > 0.05, Mann–Whitney U-test). Lower expression levels of study ncRNA were observed in patients with diagnosed lymph node metastases (N1 + N2), in comparison to those without them (N0) (Table 3).

Focus on tumor size, the highest miR-150 expression level was observed in a patient with T1 and the lowest in patients with T3 + T4; however, the highest expression of linc00673 was noticed in patient with T3 + T4 and the lowest in T2 patients (Table 3). Statistically insignificant correlations were found (p > 0.05, Kruskal–Wallis test).

### Relative expression levels of miR-150 and linc00673 in NSCLC tissue vs. serum EVs of NSCLC patients

It was found, that 57% of serum samples taken from NSCLC patients presented a downregulated miR-150 expression level in EVs but upregulated expression level of linc00673. The relative expression levels of study miRNA and lncRNA in tumor tissue were statistically significant different to those in the serum EVs of NSCLC patients (p < 0.0001, Mann–Whitney U-test). Higher expression level of miR-150 and linc00673 was noticed in tumor tissue in comparison to serum EVs (median RQ: 27.897, 47.672 and 0.656, 1.564, respectively).

### miR-150 and linc00673 expression levels in the serum EVs of NSCLC patients vs. control

NSCLC patients presented a lower miR-150 expression level in serum EVs in comparison to controls (median RQ: 0.656 and 1.087, respectively) but higher linc00673 expression level (median RQ: 1.564 and 0.920, respectively). Statistically insignificant differences in miR-150 and linc00673 expression levels were observed in the study groups (p > 0.05, Mann–Whitney U-test).

In the serum EVs of AC patients, the expression level of miR-150 was significantly lower than in controls (median RQ: 0.207 and 1.087, respectively), but the expression level of linc00673 was significantly higher (median RQ: 4.840 and 0.920, respectively) (p = 0.002, Mann–Whitney U-test). Conversely, in the serum EVs of SCC patients, the miR-150 expression level was higher compared to control (median RQ: 1.726 and 1.087, respectively) and the expression level of linc00673 was lower (median RQ: 0.580 and 0.920, respectively). Statistically insignificant differences in linc00673 and miR-150 expression levels were found between the SCC patients and control group (p > 0.05, Mann–Whitney U-test) (Fig. 2).

### miR-150 and linc00673 expression levels in the serum EVs of NSCLC patients according to biological characteristics and smoking history of study patients

A higher miR-150 expression level in serum EVs was observed in group of men than women (median RQ: 0.760 and 0.414, respectively), while women in comparison to men demonstrated a higher linc00673 expression level (median RQ: 2.414 and 1.316, respectively) (Table 4). Statistically insignificant differences in ncRNA expression levels were found between the two groups (p > 0.05, Mann–Whitney U-test).

**Table 4:** Median expression level (RQ value) of studied ncRNAs in serum EVs of NSCLC patients according to clinical and pathological characteristics.

Clinical and pathological features	miR-150 (median RQ)	p value	linc00673 (median RQ)	p value
Gender				
Female	0.414	> 0.05	2.414	> 0.05
Male	0.760		1.316	
Age				
≤ 65	0.656	> 0.05	1.564	> 0.05
> 65	0.653		1.768	
Smoking status				
Never smokers	1.116	> 0.05	0.896	> 0.05
Former smokers	0.392		2.553	
Current smokers	0.552		1.812	
Smoking history				
≤ 35 PYs	0.722	> 0.05	1.467	> 0.05
> 35 PYs	0.400		2.504	
Histopathological subtype				
SCC	1.726	**0.03**	0.580	**0.03**
AC	0.207		4.840	
AJCC staging system				
Stage I	1.108	> 0.05	0.902	> 0.05
Stage II	0.656		1.564	
Stage III	0.414		2.414	
pTNM (N)				
N0	0.722	> 0.05	1.467	> 0.05
N1 + N2	0.587		1.865	
pTNM (T)				
T1	0.414	> 0.05	2.414	> 0.05
T2	1.059		1.566	
T3 + T4	0.891		1.122	

Significant values are in bold.

Statistically significant p < 0.05.

In the serum EVs of patients aged > 65 was observed a slightly lower miR-150 expression level but higher linc00673 expression level than those aged ≤ 65 (Table 4). Statistically insignificant differences in the level of miR-150 and linc00673 expression were noticed between the two groups (p > 0.05, Mann–Whitney U-test).

Regarding the smoking status, the expression level of miR-150 in serum EVs was lower in current smokers in comparison to never smokers, but higher than in formers smokers (Table 4). Conversely, the expression level of linc00673 was higher in current smokers than in never smokers. It was however lower in comparison to former smokers. There was statistically insignificant correlation between the study groups (p > 0.05, Kruskal–Wallis test). Patients consuming > 35 PYs, demonstrated a lower expression level of miR-150 in comparison to those consuming ≤ 35 PYs (median RQ: 0.400 and 0.722, respectively), but the expression level of linc00673 was higher (median RQ: 2.504 and 1.467, respectively). Statistically insignificant differences were noticed between the two groups (p > 0.05, Mann–Whitney U-test).

### miR-150 and linc00673 expression levels in the serum EVs of NSCLC patients according to histopathological evaluation and the TNM/AJCC staging system

Significant differences in miR-150 or linc00673 expression levels were noticed regarding to the histopathological type of NSLC (p = 0.03, Mann–Whitney U-test). A higher miR-150 expression level was observed in SCC compared to AC (median RQ: 1.726 and 0.207, respectively). Conversely, linc00673 expression level was decreased in SCC compared to AC (median RQ: 0.580 and 4.840, respectively) (Table 4).

With regard to the AJCC staging system, the lowest miR-150 expression level was noticed in a patient with stage I (Table 4) and the highest in stage III. Conversely, the highest linc00673 expression level was observed in stage I and the lowest—in stage III. Statistically insignificant correlations in the expression level of any studied ncRNA were observed regarding to the AJCC stage (p > 0.05, Kruskal-Willis test).

In the serum EVs collected from NSCLC patients, a lower miR-150 expression level and higher linc00673 expression level were observed in patients with diagnosed lymph node metastases (N1 + N2) than in non-metastatic patients (N0) (Table 4), but statistically insignificant differences were noticed (p > 0.05, Mann–Whitney U-test).

Statistically insignificant correlations were found between the study ncRNAs expression level and tumor size (p > 0.05, Kruskal–Wallis test). The highest miR-150 expression was observed in a patient with T2 and the lowest in patients with T1 (median RQ: 0.972 and 0.903 respectively). Conversely the highest linc00673 expression level was observed in a patient with T1 and the lowest in patients with T3 (Table 4).

### Correlation between the expression levels of the studied genes and ncRNAs in NSCLC patients

A significant positive correlation was found between the expression level of CCR6 and miR-150 in tumor tissue (p = 0.035, rho = 0.386, Spearman’s rank correlation). In addition, a significant positive correlation was observed between the expression level of CCR6 and linc00673 in tumor tissue (p = 0.01, rho = 0.466, Spearman’s rank correlation). Furthermore, a significant negative correlation was noticed between the expression level of linc00673 in tumor tissue and serum EVs of NSCLC patients (p = 0.028, rho = − 0.402, Spearman’s rank correlation). No significant correlation was found between the expression level of miR-150 and linc00673 either in tumor tissue or serum EVs of NSCLC patients.

### Receiver operating characteristic (ROC) curve analyses

**Figure 3:**
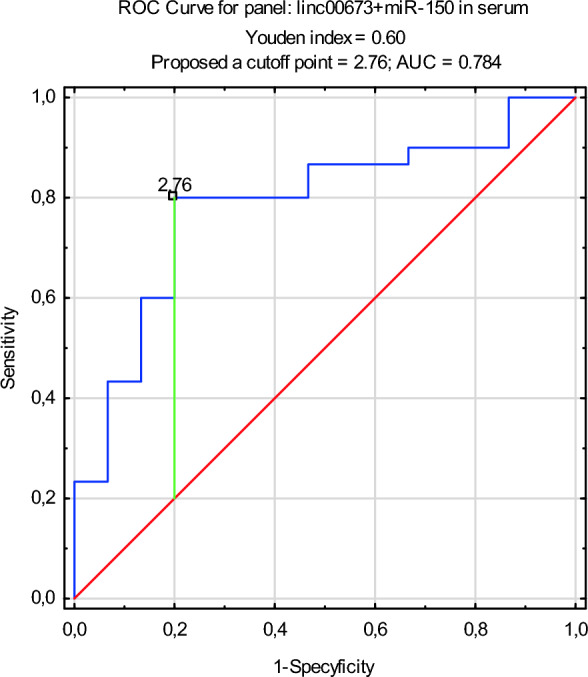
Receiver operating characteristic (ROC) curve analysis for serum EVs combined ncRNAs diagnostic potential in differentiating NSCLC and control.

ROC curve analyses were performed to calculate the diagnostic value of the studied ncRNAs in serum of NSCLC patients compared to healthy control group. The expression level of miR-150 and linc00673 in serum EVs of NSCLC patients were analyzed as combined (in a panel). AUC for studied ncRNA was 0.784. An optimal cutoff point for this panel was indicated at 2.76 with a sensitivity of 80% and a specificity of 80% (p = 0.0001) (Fig. 3).

## Discussion

The survival rate of NSCLC patients depends on the stage of the cancer development at the period of diagnosis. Unfortunately, patients are often diagnosed in the advance stage of the disease with metastases to regional lymph nodes or to distant organs. According to a report of the American Cancer Society, (ACS), the survival ratio of patients’ in the advanced stage of disease is only 5.8%^29^. Therefore, there is an urgent necessity to develop biomarkers useful for early stage tumor diagnosis and for monitoring tumor progression, which can be served for quick, non-invasive diagnostics.

Considering the molecular mechanism of carcinogenesis in the lung, it was documented that the significant role in development and progression of NSCLC serves chemokines, a small-molecule peptides that are classified as cytokines with chemotactic properties^30,31^. The activity of chemokines and their influence on target cells is closely related to the stimulation of their G-protein coupled 7-transmembrane receptors^32^. It was revealed the chemokine CCL20, which specifically bind to CCR6 plays a significant role in development of NSCLC by regulation of lung cancer cell proliferation, apoptosis, migration, EMT, as well as they are involving in modification of the immune response to cancer^5,9,33,34^.

Change in the expression level of mRNA *CCL20* and CCR6 in the lung during tumorigenesis has been proven by other researchers^5^. Also in our study was proved a change in the expression of *CCL20* and *CCR6* mRNA in lung cancer tissue. We revealed a higher expression level of *CCL20* mRNA and lower expression level of *CCR6* mRNA in NSCLC samples in comparison to control tissue. Our study showed that *CCL20* mRNA expression level was upregulated (RQ > 1) in 47% of NSCLC tissue samples and in 23% of control tissues, while upregulated expression level of *CCR6* mRNA was noticed in 97% NSCLC tissue samples and all (100%) of control samples. A higher level of *CCL20* mRNA and lower level of *CCR6* mRNA expression in tumor tissue was also noticed in previous studies, which is consistent with our results^5^. Our investigation confirmed that CCL20 is a key chemokine which promotes NSCLC development. However, during tumorigenesis, the expression level of *CCR6* mRNA decreased, in contrast to the expression level of *CCR7* mRNA. We have also demonstrated this relationship in previous studies^35^. We confirmed that specific and differential pattern of *CCR6*, *CCR7* mRNA expression levels was manifested in NSCLC tumor cells. Results of our study suggest that a loss of *CCR6* expression and an increase in *CCR7* expression may allow cancer cells to migrate to lymph nodes and lead to development of metastasis. Cancer cell migration is highly similar to leukocyte transport, which is mainly regulated by chemokines and their receptors^36^. In response to inflammatory stimuli, immune cells lose expression of CCR6, while CCR7 expression increases, leading to their migration to lymph nodes via a gradient of the CCL19 and CCL21 chemokines, specific ligands for the CCR7 receptor^36,37^. Interestingly, the decreased expression level of *CCR6* mRNA, but increased expression of *CCR7* mRNA was noticed also in a study of Wang et al. in metastatic primary tumor of the head and neck carcinomas^37^, which provides evidence of common key mechanisms of chemokine-mediated metastasis in cancer.

The expression levels of mRNA studies genes were also analyzed in our study according to the size of the tumor (feature T according to the TNM staging). We noticed that the level of *CCR6* mRNA expression negatively correlated with the growth of the tumor; however, the expression level of *CCL20* mRNA was higher in larger tumors (T3 + T4 *vs.* T1 and T2). Unfortunately, there are only a few studies on this issue^7,33,34,38^, which is not enough to explore the topic. However, Minamiya et al. obtained similar results. They demonstrated a lower level of *CCR6* mRNA expression in a greater percentage of patients with larger NSCLC tumors (> 30 mm) than with smaller ones (≤ 30 mm)^38^. In contrary, Kirshberg et al. showed that the expression level of *CCR6* mRNA increases with tumor growth (T feature according to the TNM staging). Nevertheless, we must point out that their observations were based on a small number of samples (n = 3)^7^. Interestingly, the in vitro study on NSCLC cell lines (A549) provides a better understanding of the chemokine-dependent mechanism of tumor progression^6,33^. Zhang et al. documented that activation of CCR6 on the surface of cancer cells by CCL20 induces tumor cell proliferation via the PI3K and ERK signaling pathway^33^. This mechanism is also involved in the regulation of programmed cell death resulting in tumor growth^34^.

On the other hand, an in vitro study conducted by Sutherland et al. revealed that a decreased expression level of *CCR6* mRNA may be associated with a more metastatic phenotype of NSCLC^39^. These results suggest that a lower expression level of *CCR6* mRNA in NSCLC patients with a larger tumors size (T feature) may be a selected feature of cancer cells acquiring the ability to emigrate from peripheral mucosal sites where CCL20 is highly expressed. This hypothesis is not supported by our research results because we showed that the expression level of *CCR6* mRNA was higher in NSCLC patients with diagnosed lymph nodes metastasis (N1 + N2) compared to non-metastatic cancer patients (N0). This finding may suggest that cancer cells after invading out of the primary tumor, can increase expression of CCR6 to allow their migration to a lymph node and distant organs, especially to the adrenal gland^40^. The proposed concept is confirmed by the observed high expression of CCL20 on adrenal gland cells^40^. A study of Raynaud et al. demonstrated that in adrenal metastasis originating from a primary lung tumor, the expression level of *CCR6* mRNA was higher compared to that observed in lung primary tumor^40^. The increase of metastatic potential of CCR6-expressing lung cancer cells may result from a decrease in local production of CCL20 by the tumor cells themselves (and possibly also by stromal cells), which facilitates migration of tumor cells away from their original site. Furthermore, our analysis of study gene expression level according to the stage of cancer development (AJCC staging) revealed a higher expression level of *CCR6* mRNA and lower expression level of *CCL20* mRNA in patients with the advanced stage of cancer compared to patients with early stage of the disease, without metastasis to lymph nodes (stage III *vs.* stage I).

The airway epithelium is the main producer of CCL20, and tobacco smoking is one of factors that increase secretion of this chemokine^5,41^. Moreover, tobacco smoking possess the major risk factor for lung cancer and may be responsible for up-regulation of CCL20 expression at both mRNA and protein levels^5^. Interestingly, an in vitro study of Wang et al. demonstrated that the expression level of *CCL20* mRNA either in normal human lung epithelial cell line (16HBE) or lung cancer cell lines (A549) is induced by NNK, a key carcinogen of tobacco^5^. Moreover, the clinical study of Wang et al. revealed that in the group of smokers the expression level of *CCL20* mRNA was significantly higher than in the non-smoker group^5^. Our results are confirmed with theirs observations. Moreover, in our study we also observed that in group of former smokers the expression level of *CCL20* mRNA was higher than in non-smokers and smokers group. The higher expression level of *CCL20* mRNA in former smokers compared to patients who still smoke tobacco may have resulted from inhibited exposure to nicotine that is an important immunosuppressive constituent of cigarette smoke^42,43^. Recent studies by Valdez-Miramontes et al. revealed that nicotine reduces the immunoexpression level of chemokines in airway epithelial cells such as CCL5, CXCL10, as well as interleukin 6 (IL-6), being a factor that stimulates the expression of CCL20^44,45^. We also observed that the level of *CCL20* mRNA expression decreased as smoking intensity increased, but increased the expression level of mRNA *CCR6*. Our study also showed a positive correlation between the expression level of CCR6 mRNA and the smoking status of patients with NSCLC (the lowest expression level of this gene was observed among non-smokers, subsequently higher in former smokers and the highest in current smokers). Additionally, we observed a positive correlation between the *CCR6* mRNA expression level in tumor tissue of smokers and smoking history (amount of PYs). It should be pointed out that it is the first such report of *CCR6* mRNA expression level change in tumor tissue of NSCLC patients related to smoking. Increased levels of *CCR6* expression in tumor tissue may be caused by infiltration of regulatory T cells (Tregs), which are known to inhibit antitumor effects^46^. A study of Zhang et al. demonstrated that the higher level of Tregs infiltration to tumor sites is associated with shorter survival time of NSCLC patients and poorer response to chemotherapy^46^.

In our study we also focus on assessment of mRNA expression level of study genes according to the histopathological type of cancer. A higher *CCR6* and *CCL20* mRNA expression level was observed in the AC group compared to the SCC group, which may be responsible for stronger biological aggressiveness of this histopathological type of lung cancer. Hippe et al. revealed that the EGFR/Ras activation increases the expression level of *CCL20* and promotes CCL20 production in a large group of tumor cell lines, among others breast cancer, melanoma, head and neck squamous cell carcinoma, changing cells from a resting phenotype into a proliferative and migratory one^47^. In the case of NSCLC, the activation of EGFR/Ras signaling pathway via mutations occurs more frequently in AC, especially in smokers^48,49^.

Liquid biopsy is a modern diagnostic method in clinical oncology, which is becoming very popular because it allows to identify, investigate, and monitor cancer cells in body fluids such as blood, serum, plasma or urine^50^. Liquid biopsy in relation to tumor biopsy is more easily accessible, in most cases painless, and more comprehensive for assessing heterogeneity within the tumor^51^. This method relies on the analysis of materials extrected by tumor into circulation, such as circulating tumor cells (CTCs), cell-free nucleic acids (cfNAs), and extracellular vesicles (EVs)^52^.The discovery of highly sensitive and accurate biomarkers by liquid biopsy could be a promising approach while making a non-invasive diagnosis of lung cancer prior to clinical manifestation of the disease, which is considered an effective strategy for reducing cancer mortality. We analyzed the expression level of study ncRNA(miRNA and lncRNA) in liquid biopsy (serum EVs) of NSCLC patients compared to the control group.

MiRNAs have been shown to exert complex effects on lung cancer development and progression, including regulation of cancer growth, angiogenesis and metastasis, domination of host immune responses and remodeling of the tumor microenvironment^53–56^. In recent years, miRNAs have been revealed as a valuable diagnostic biomarker of lung cancer and also a key factors for predicting the prognosis of disease^12,57^. In our study, we examined the potential for changes in miR-150 expression level in relation to the diagnostic practice. We focus on this molecule because it was revealed that miR-150 is engaged in development of solid tumors, such as breast cancer gastric cancer, hepatocellular carcinoma and also lung cancer^58^. To date, there are few studies focusing on the evaluation of miR-150 expression levels in the lung tissue of NSCLC patients^17,56^. These studies documented increasing expression of this miRNA in NSCLC tissue compared to control tissue, indicating its oncogenic function^15,59^. However, there are data confirming the suppressive role of miR-150 in NSCLC, where its expression level decreases during carcinogenesis^60^. In our study, we showed that the expression level of miR-150 was significantly reduced compared to control tissue. Similarly to our observations, the decreased expression level of miR-150 was observed in the serum of NSCLC patients by Zaporozhchenko et al.^65^. However, in contrast to results we obtained, a study of Jiang et al. demonstrated an increased expression of this miRNA in the serum of NSCLC patients compared to the control group^66^. In our research, we assessed the expression of miR-150 in relation to linc00673 on the base of data obtained by Lu et al. in their research^23^. The authors revealed a reduced miR-150 expression level in cancer cells during tumorigenesis by its binding to linc00673. They documented that the decreased expression level of miR-150 was correlated with the increased expression level of linc00673^23^. It was also demonstrated that in the NSCLC cell line (A549), miR-150/linc00673 binding mechanism promotes cell proliferation, migration, invasion and EMT^23^. Our study focus on linc00673 demonstrated that the expression level of linc00673 was increased in tumor tissue compared to control tissue. Other authors confirmed upregulation of linc00673 in tumor tissue and identified the linc00673 as an oncogene in NSCLC^24,25^, which is in line with our results. Moreover, according to our knowledge, we were the first who assessed the expression level of linc00673 in the serum of NSCLC patients.

A study of Dai et al. demonstrated that the expression level of miR-150-5p may potentially serve as a biomarker for predicting NSCLC progression. They revealed that NSCLC patient group with a lower expression level of miR-150-5p had a shorter progression-free time, overall survival time and a higher relapse rate^18^. Our analyses revealed a negative correlation between the stage of cancer development according to the AJCC classification and the expression level of miR-150 in tumor tissue. The expression level of linc00673 did not reveal a correlation; however, its highest expression level was noticed in stage III. Sun et al. in the their study confirmed a lower expression level of miR-150 in NSCLC tumor from patient in advanced stages of disease (stages III + IV according to the AJCC staging) compared to those in earlier stages (stages I + II) and this observation corresponds to ours. Shi et al. also showed a positive association between the expression level of linc00673 in tumor tissue and progression of NSCLC disease (AJCC staging)^25,60^. Furthermore, our analysis revealed a negative correlation between the tumor size (feature T according to the pTNM staging) and the expression level of miR-150 in NSCLC tumor tissue. In contrast, the expression level of linc00673 did not reveal such a correlation but its highest expression level was noticed in T3 + T4., Sun et al. made an observation which corresponded to our findings as they demonstrated that the expression level of miR-150 was significantly lower in larger tumors (size ≥ 3 cm diameter) rather than in smaller ones (< 3 cm diameter) and a study of Shi et al. revealed a higher expression level of linc00673 in larger tumors (≥ 5 cm diameter) than in smaller ones (< 5 cm diameter)^25,60^.

The expression level of tested ncRNA in tumor tissue of NSCLC patients was assessed on the basis of the lymph node involvement status (feature N according to the pTNM staging). NSCLC tumor tissue of patients with diagnosed metastases (N1 + N2) revealed a lower expression level of mir-150 and linc00673 compared to tissue of patients without metastases (N0). Like us, Sun et al. demonstrated that decreased mir-150 expression level was related to presence of lymph node metastasis. However, a study of Shi et al. showed higher expression level of linc00673 positively correlated with presence of lymph node metastasis^26,56^. It should be reflected whether the elevated linc00673 expression level, observed in N0 patients in our study, may indicate the presence of micro-metastases in the lymph nodes, which are not taken into account by current staging criteria during assessments of lung cancer^61^.

Furthermore, our analyses of studied ncRNAs expression level in the serum EVs of NSCLC patients with regards to the clinical stage of cancer revealed a negative correlation with the miR-150 expression level and positive correlation with linc00673 expression level according to the stage of tumor disease. Our study is also the first one to show that evaluation of the expression level of miR-150 and linc00673 in the serum EVs of a NSCLC patients may potentially serve as a non-invasive biomarker of tumor progression. Moreover, our analyses showed a negative correlation between tumor size and the expression level of linc00673, but did not reveal a correlation with the expression level of miR-150. However, its lowest expression level was observed in T1. Furthermore, our study demonstrated a lower expression level of mir-150 and a higher expression level of linc00673 in the serum EVs of NSCLC patients with detected lymph node metastases compared to those without them. Such studies have not been conducted in a group of NSCLC patients before, though, the reduced serum level of miR-150 expression correlated with the incidence of metastases of colorectal cancer^61^.

Regarding the histopathological type of cancer, the expression level of miR-150 in SCC tumor tissue was lower compared to AC; however, the expression level of linc00673 was higher. Consistent to our results A lower expression level of miR-150 in SCC tumor tissue was noticed in a study by Sun et al. and their findings correspond to ours. Yet, unlike us, Shi et al. revealed that more SCC patients demonstrated a low expression level of linc00673^25,60^. A study conducted by Misono et al. revealed that in AC, miR-150 plays a suppressive function as it inhibits expression level of *TNS-4,* an important oncogene^62^. A decreased expression level of this miRNA in tumor was strongly related to shorter disease-free survival and poorer prognosis of AC patients. A study of Suetsugu et al. also showed the suppressor function of miR-150 during SCC carcinogenesis and identified *MMP14* mRNA as a target of miR-150 regulation in SCC cells^63^. Zheng et al. confirmed that high immunoexpression level of MMP14 predicts a poor prognosis of SCC patients^64^.

It should be pointed out that our study is the first one to demonstrate differences in the miR-150 and linc00673 expression levels in the serum EVs of AC and SCC. We observed statistically significantly higher levels of miR-150 expression, but lower levels of linc00673 expression in SCC compared to AC, which indicates that miR-150 and linc00673 may serve as non-invasive biomarkers for differentiation of these two histopathological types of NSCLC patients. Furthermore, our study revealed a significantly lower expression level of miR-150 in the serum EVs of AC patients, but a higher expression level of linc00673 compared to the control group, which indicates that assessment of study ncRNAs may be used as a diagnostic biomarker in liquid biopsy in this group of patients. The serum EVs of SCC patients and controls did not reveal statistically significant differences in the expression level of miR-150 and linc00673.

Our analysis did not reveal any significant association between the expression level of study ncRNAs in tumor tissue and the smoking status. In current smokers, the expression level of miR-150 and linc00673 was higher compared to that in never smokers and also former smokers. In patients who smoked > 35 PYs, the expression level of miR-150 was higher but the expression level of linc00673 was lower than in those who smoked ≤ 35 PYs. As reported in an in vitro study by Xue et al. a high level of miR-150 expression may be associated with the acquisition of human bronchial epithelial cells resistance to apoptosis, prompted by cigarette smoke extract and reduced activity of caspase 3, suggesting a protective effect of miR-150^65^. Contrary to our findings a study of Sun et al. revealed that current or ever smokers demonstrated a significantly lower expression level of miR-150 compared to never smokers. Shi et al. in turn showed a negative correlation of linc00673 expression level with the smoking status^25,60^. A recent study of Wu et al. revealed that benzo(a)pyrene (BaP), being a major toxicant which is abundant in tobacco smoking, significantly elevates linc00673 expression level in NSCLC cancer cells and promotes their migration, invasion and EMT^66^.

We were also the first to assess changes in the expression level of miR-150 and linc00673 in the serum EVs of NSCLC patients according to the smoking status. The analyses revealed that the expression level of miR-150 in the serum EVs of current smokers was lower in comparison to never smokers but higher than in former smokers. Furthermore, a negative correlation between the miR-150 expression level in the serum EVs of smokers and smoking history (amount of PYs) was observed. In turn, a higher expression level of linc00673 was observed in the serum EVs of current smokers compared to never smokers but lower than in former smokers. Moreover, the serum EVs linc00673 expression level showed a positive correlation with smoking history.

No significant correlation was found between the expression level of miR-150 and linc00673 either in tumor tissue or serum EVs of NSCLC patients. However, study of Lu et al. revealed a significantly inverse correlation between expression level of linc00673 and miR-150 in NSCLC tissues^22^.

Using the ROC curve analysis for miR-150 and linc00673 separately, we did not found diagnostic value of the studied ncRNAs in serum of NSCLC patients compared to healthy control group. The combination of miR-150 and linc00673 shows good test accuracy for NSCLC^67^. Thus, these two ncRNAs in serum EVs may have potential for further evaluation as biomarkers in the early diagnosis of NSCLC.

The fact that in our single-center studies only small groups of patients were included seem to be a significant limitation of our research. The small number of patients studied is probably the main reason why relatively few statistically significant results were obtained in our research, regarding the relationship between the expression level of *CCR6/CCL20* mRNA, study ncRNAs, and clinical features of patients or histopathological diagnosis.

## Conclusions

Our study confirmed that smoking significantly changes the level of *CCL20* mRNA expression in tumor tissue in NSCLC patients. It was also shown that the level of expression of *CCL20* mRNA in the tumor tissue as well as miR-150 and linc00673 in the serum EVs can be used as a diagnostic marker differentiating adenocarcinoma from squamous cell carcinoma. Moreover, our analyzes showed a correlation between the expression level of linc000673 and miR-150 with disease progression, indicating the oncogenic function of linc000673 and the suppressor function of miR-150 in development of NSCLC. Changes in the expression level of linc000673 and miR-150 in the serum EVs of patients with NSCLC may be a potential non-invasive diagnostic marker with a negative prognostic value.

## Data Availability

The datasets used and/or analysed during the current study available from the corresponding author on reasonable request.
